# First clinical case report of local microinjection of autologous fat and adipose-derived stromal vascular fraction for perianal fistula in Crohn’s disease

**DOI:** 10.1186/s13287-017-0736-6

**Published:** 2018-01-10

**Authors:** Cécile Philandrianos, Mélanie Serrero, Fanny Grimaud, Jérémy Magalon, Carine Visée, Mélanie Velier, Pauline Francois, Pierre Orsoni, Guy Magalon, Jean-Charles Grimaud, Ariadne Desjeux, Julie Véran, Florence Sabatier

**Affiliations:** 1 Plastic Surgery Department, Assistance Publique Hôpitaux de Marseille (APHM), Aix Marseille University, Marseille, France; 2 Gastroenterology Department, APHM, Aix Marseille University, Marseille, France; 3 Culture and Cell Therapy Laboratory, INSERM CBT-1409, APHM, Aix Marseille University, Marseille, France; 4 Digestive Surgery Department, APHM, Aix Marseille University, Marseille, France; 5 Vascular Research Center Marseille (VRCM), Aix Marseille University, INSERM UMR 1076, Marseille, France

## Abstract

Mesenchymal stem cell therapy is a promising treatment for perianal Crohn’s fistulas refractory to conventional therapy, which are an extremely morbid complication and a true therapeutic challenge. Autologous adipose-derived stromal vascular fraction (ADSVF) is an easily accessible source of cells with angiogenic, anti-inflammatory, immunomodulatory, and regenerative properties. Here, we describe a case involving a patient with severe perianal Crohn’s fistulas refractory to the best medical and surgical practices who received local treatment with ADSVF and microfat. This patient was first examined under anesthesia with drainage via seton placement; 1 week later, on a single day, he underwent adipose tissue extraction, ADSVF and microfat preparation, and the local injection of 14 ml of microfat and approximately 20 million viable ADSVF cells into the soft tissue around the fistulas. No serious adverse events were observed. At the first endpoint at 12 weeks, the fistula had clinically healed with complete re-epithelialization of all external openings; no fistula tract was detected on magnetic resonance imaging, confirming this finding. This good clinical outcome was sustained at 48 weeks and was associated with a reduction in the severity of perianal disease and an improvement in quality of life. The current case highlights the therapeutic potential of a new cellular treatment for Crohn’s patients with refractory perianal fistulas based on the innovative hypothesis that the combined action of ADSVF in association with the trophic characteristics of a microfat graft could be beneficial for this condition.

Trial registration: EudraCT number 201325, NCT02520843. Registered on 5 August 2015

## Background

Crohn's disease is a transmural chronic inflammation that can affect any part of the digestive tract, ranging “from the mouth to the anus”. Perianal fistulas are frequent, occurring in almost one-third of Crohn’s patients, and the incidence of this condition is increasing [1]. In addition, the various problems induced by fistulas, such as pain, purulent discharge, abscess formation, and occasional anal incontinence, deeply affect patients’ quality of life. Thus, anoperineal lesions represent a true clinical challenge and are currently difficult to treat despite a large therapeutic arsenal [2]. In cases involving such lesions, a combined surgical and medical treatment approach is required. The aim of surgical management is to eradicate the fistulous tract while maintaining continence. Autologous adipose-derived stromal vascular fraction (ADSVF) is recognized as an easily accessible source of cells with angiogenic, anti-inflammatory, immunomodulatory, and regenerative properties [3]. ADSVF, combined with microfat grafting that provides volumizing and trophic effects [4, 5], could have beneficial effects for the treatment of Crohn’s fistulas. We obtained approval from the appropriate French regulatory agency and ethics committee for our proposal to deliver a combined microfat and ADSVF treatment to ten patients who presented with recurrent perianal fistulas in the context of Crohn’s disease (NCT 02520843). Here, we present the first case of a patient who reached the 48-week follow-up time point.

## Materials and methods

### Patient

After providing written informed consent, a 34-year-old man was included in the ADICROHN prospective phase I clinical trial (EudraCT number: 201325) registered at clinicaltrials.gov. He had presented with a complex perianal fistula associated with Crohn’s disease that was objectively assessed via a clinical examination under general anesthesia and magnetic resonance imaging (MRI) in accordance with recognized clinical, endoscopic, and histological criteria. The fistula was intersphincteral at the 7 o’clock position (Fig. 2; MRI at baseline) and presented with three external openings. The fistula was refractory to conventional medical and surgical treatments. The patient had been receiving long-standing treatment with infliximab 10 mg/kg every 8 weeks. His body mass index was 22 kg/m^2^. One week before the investigational product was administered, he underwent a fistula preparation visit that included an examination under anesthesia, fistula curettage, and seton placement as clinically indicated.

### Assessments

The primary safety endpoint was the assessment of inflammatory clinical signs at weeks 0, 1, 2, 6, 12, 16, and 48, with assessments confirmed via routine laboratory measurements at weeks 0, 12, and 48. The patient was monitored for potential adverse events at each study visit.

The secondary endpoints were evaluations of efficacy, defined as the results of clinical assessments at weeks 1, 2, 6, 12, and 16 of the closure of all external openings that were draining at baseline despite gentle finger compression; these findings were confirmed via pelvic MRI scans indicating the absence of collections > 2 cm for treated perianal fistulas at weeks 12 and 48. At baseline and at all study visits, the severity of perianal Crohn’s disease was assessed using the Perianal Disease Activity Index (PDAI; scored from 0–20, with a higher score indicating more severe disease), the quality of life was measured using the Short Inflammatory Bowel Disease Questionnaire (SIBDQ; scored from 10–70, with higher scores indicating better quality of life), and the activity of luminal Crohn’s disease was assessed using the Crohn’s Disease Activity Index (CDAI; scored from 0–600, with the cut-off value between remission and active disease traditionally defined to be 150 points).

#### Surgical procedure

**Fig. 1:**
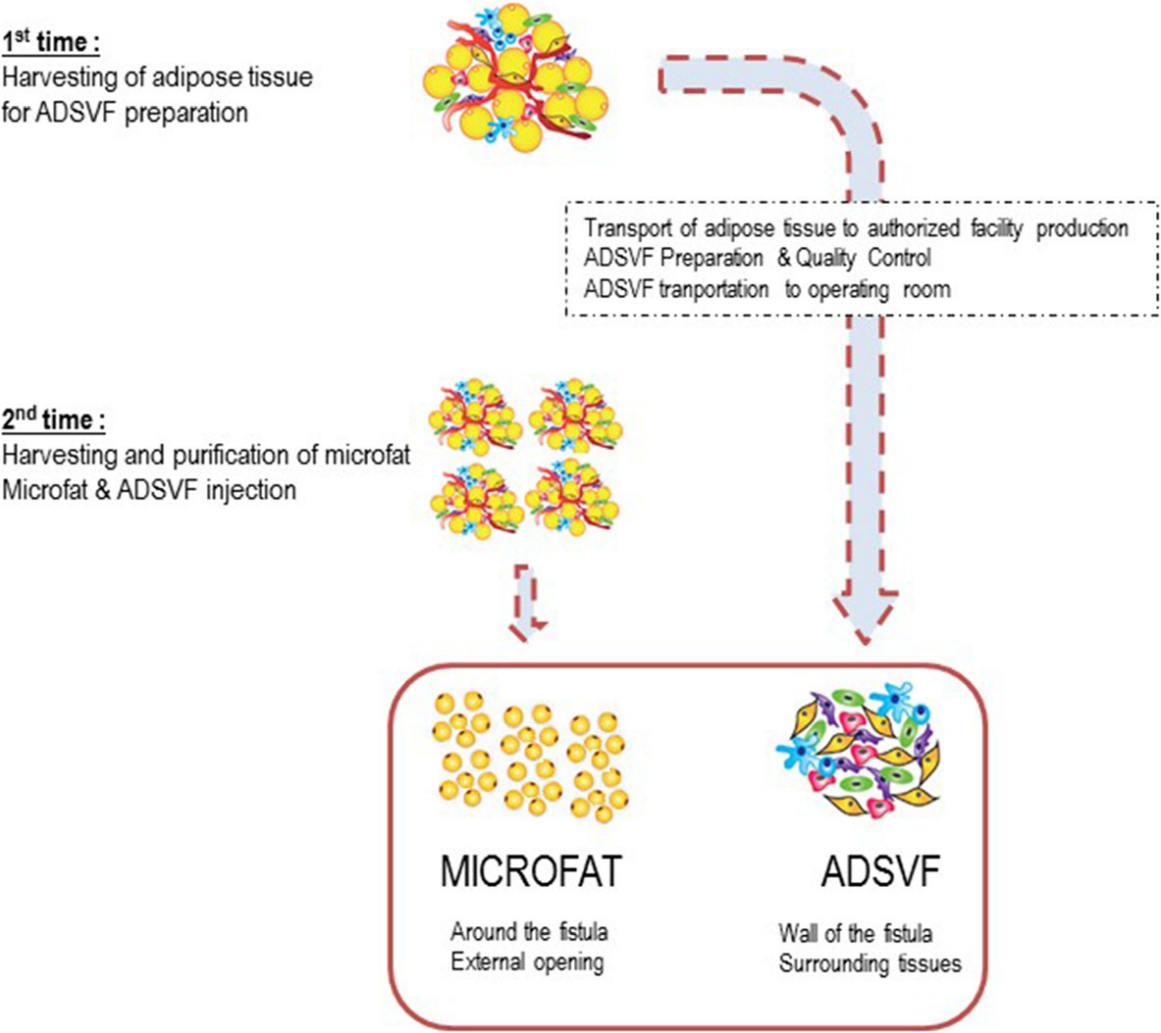
Representation of the two surgical times for the 1-day procedure. *ADSVF* adipose-derived stromal vascular fraction

The surgical procedure requires the performance of two consecutive surgeries on the same day under general anesthesia (Fig. 1). The first surgery consists of lipoaspiration to obtain ADSVF. Adipose tissue was harvested with a 10-ml syringe in a closed circuit, a 3-mm Khouri cannula, and a 250-ml collection bag. A total of 150 ml of lipoaspirate was collected, transported to the registered Cell Therapy Unit of La Conception University Hospital, and transferred to a Celution 800/CRS system (Cytori Therapeutics, Inc., San Diego, CA, USA). Collected lipoaspirate was washed and enzymatically digested using good manufacturing practice (GMP)-grade reagents. Cells were concentrated, washed, aseptically recovered, and resuspended in 6 ml Ringer’s lactate solution; 1 ml of the resulting suspension was used for sterility testing and biological characterization. Total viable nucleated cell recovery and cell viability were determined using a Nucleocounter NC-100 instrument (ChemoMetec, Denmark). Cellular components within isolated ADSVF samples were identified using flow cytometry analysis (with a Beckman Navios instrument; Beckman Coulter, Miami, FL, USA) conducted using a panel of cell surface makers in accordance with recommendations of the International Federation for Adipose Therapeutics and Science (IFATS) and the International Society for Cellular Therapy (ISCT).

The second surgery consisted of microfat harvesting and the reinjection procedure for both microfat and ADSVF. Liposuction was performed using a 2-mm st’RIM cannula (Thiebaud Biomedical Devices, Margencel, France) connected to a 10-ml syringe. The harvested fat was directly injected into a closed-circuit Puregraft 50-ml system filtration pocket (Puregraft, San Diego, CA, USA). The Puregraft system allows for the purification of adipose tissue via the elimination of excess fluid, the lipid phase, blood cells, and fragments via membrane filtration in a sterile environment. The quantity of harvested fat was approximately 50 ml, which enabled the acquisition of a final volume of 14 ml of ready-to-inject pure fat. The cleaned adipose tissue was directly retrieved by connecting the Puregraft system to 1-ml syringes to permit precise reinjection around the fistula.

Fistula treatment requires the removal of the seton drainage. The fistula tract was identified, and careful curettage was performed. The internal opening was closed with resorbable sutures. Microfat was injected around the fistula using a 21 G cannula from the st’RIM device to fill adjacent tissues and collapse the fistula. A 25 G needle was then used to inject ADSVF into the fistula wall and surrounding tissue. Finally, the external opening was obliterated via microfat injection into periorifice tissue.

## Results

Microfat and ADSVF characteristics are summarized in Table 1.

**Table 1:** Characteristics of the adipose-derived stromal vascular fraction and microfat biological parameters

**Stromal vascular fraction**	
Adipose tissue harvested (cm^3^)	190
Volume (ml)	5
Number of viable nucleated cells (millions) obtained (before quality control)	35.3
Recovery rate (viable nucleated cells/cm^3^ adipose tissue)	186 000
Number of viable nucleated cells (millions) injected (after quality control)	22.8
Viability (%)	82.8
Leukocytes (%)	37.4
*Macrophages/monocytes (%)*	22.9
*Lymphocytes (%)*	12.5
*Neutrophils (%)*	2.0
Transitional cells (%)	11.2
Endothelial progenitors cells (%)	4.3
Pericytes (%)	8.5
Mesenchymal stem cells (%)	38.6
**Microfat**	
Adipose tissue harvested (cm^3^)	50
Adipose tissue injected (cm^3^)	14

With respect to the primary safety endpoint, the patient did not show inflammatory clinical signs at any of his study visits (at weeks 0, 1, 2, 6, 12, 16, and 48). This result was confirmed by routine laboratory measurements at weeks 0, 12, and 48.

No severe adverse events linked to cellular product administration were reported during the study period. The patient did not have any infectious complications or exhibit intolerance to cellular treatment. No sign of fecal incontinence was detected. The only side effect was moderate pain at the lipoaspiration site, which was linked to fat harvesting and was rapidly resolved under simple oral analgesia (with paracetamol and tramadol). The patient presented with a cutaneous reaction secondary to anesthetic drugs after an allergic investigation on a post-operative day.

**Fig. 2:**
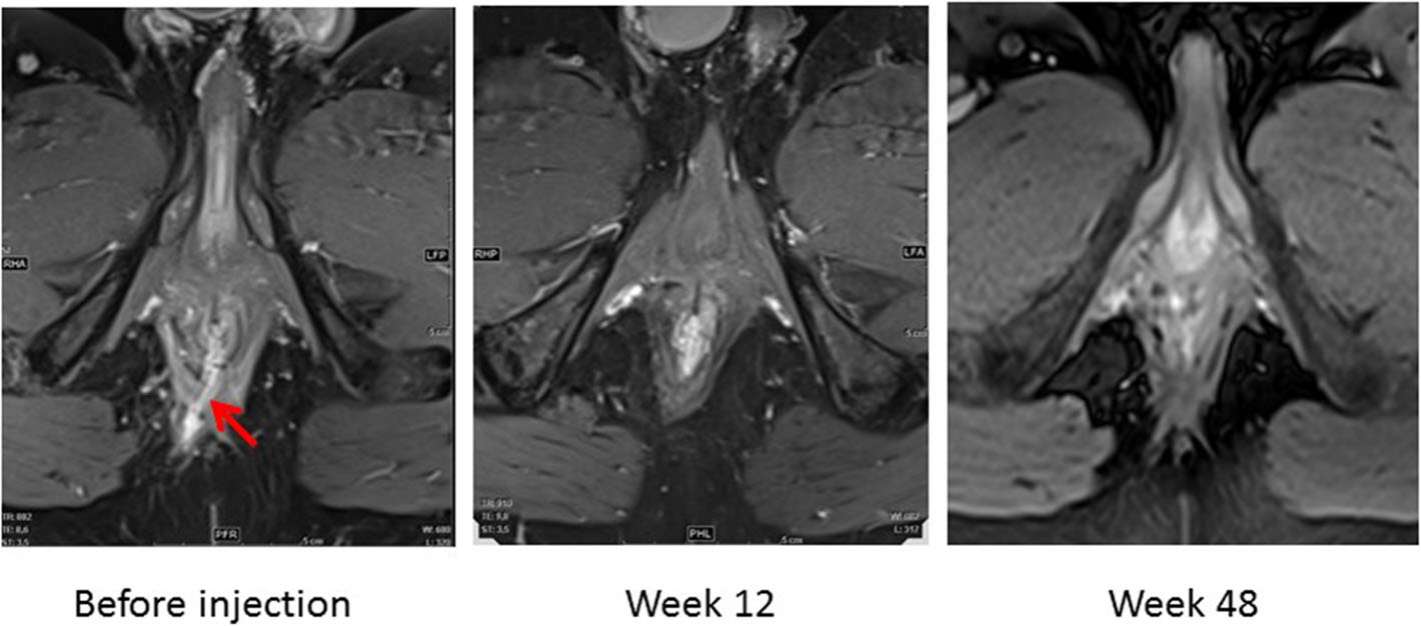
MRI images at baseline, 12 weeks, and 48 weeks. These perianal examinations of MRI results generated using T1 post-contrast (gadolinium) sequences show the disappearance of the fistula tract (red arrow) at week 12, which was confirmed at week 48, indicating complete healing of the fistula

**Fig. 3:**
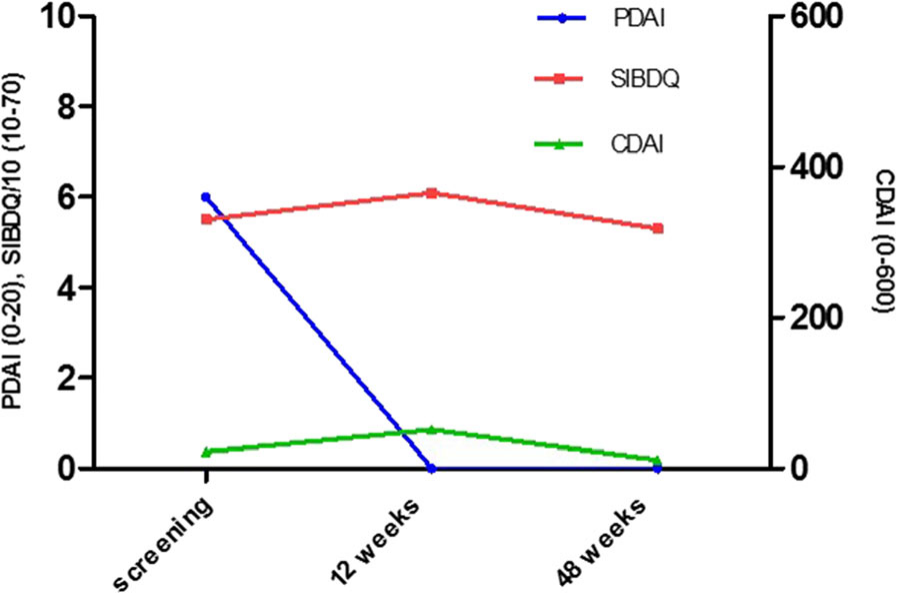
Evolution of the PDAI, SIBDQ, and CDAI scores from before screening to 12 and 48 weeks after the procedure. *CDAI* Crohn’s Disease Activity Index, *PDAI* Perianal Disease Activity Index, *SIBDQ* Short Inflammatory Bowel Disease Questionnaire

With respect to treatment efficacy, the fistula was clinically healed with complete re-epithelialization of all external openings at the first endpoint at 12 weeks; no fistula tract was detected on MRI, confirming this result (Fig. 2). The patient’s PDAI score decreased from 6 at baseline to 0 at week 12; this decrease was associated with improvement in quality of life assessed using the SIBDQ. These results were sustained through week 48; at this time, persistence of the healing of external fistula openings and the absence of a fistula tract on MRI were observed, and a PDAI score of 0 had been maintained. Despite the patient’s promising clinical response to the tested treatment, his SIBDQ score was not improved, a result that can primarily be attributed to the emotional subdomain. The activity of luminal Crohn’s disease was stable and ≤ 150 throughout the study (Fig. 3).

## Discussion

Perianal fistulas are a common complication of Crohn’s disease and are estimated to affect up to 28% of Crohn’s patients during the first two decades after diagnosis [6, 7]. They severely impair patients’ quality of life and cause substantial morbidity [8]. At present, the only approved drug for this complication is the anti-TNF monoclonal antibody infliximab. However, approximately 70–80% of these fistulas are complex fistulas that are particularly refractory to conventional medical treatment.

Recently, stem cell therapies that have mainly used expanded mesenchymal stem cells (MSCs) have shown promising effects [9]. In a phase 3 randomized, double-blind controlled trial, Panés et al. showed that a significantly greater proportion of patients achieved combined remission at 24 weeks after treatment with allogeneic adipose-derived MSCs than with treatment with placebo [10]. The efficacy of local injections of increasing doses of allogeneic bone marrow-derived MSCs was shown by complete healing at 12 weeks in seven of fifteen patients who received MSCs, compared with two of six patients who received a placebo [11]. Only one case series has compared the use of ADSVF with the use of expanded MSCs. In that series, five patients received autologous MSCs derived from adipose tissue, and four patients received ADSVF cells. The results indicated that strategies based on cell expansion prior to implantation might be more effective than those involving the direct use of ADSVF isolated from a lipoaspirate sample [12].

From a regulatory point of view, the aforementioned therapies fall under the framework of directive 1394/2007 from the European Parliament and the European Council, which described a new category of health products referred to as “advanced therapy medicinal products” (ATMPs). As ATMP production must occur in accordance with pharmaceutical industry GMP, production costs have been significantly increased. Consequently, the use of ATMPs will barely be sustainable for public institutions.

In fact, 10 years after the publication of the above regulation, few of these therapies have received marketing authorization. However, although 2 to 3 weeks of expansion are needed to produce expanded MSCs, ADSVF cells can be manufactured within a few hours, allowing for lipoaspiration and reinjection to be performed on the same day. This point should be considered from a cost-effectiveness perspective.

This report provides the first description of a therapeutic strategy that combines the volumizing use of microfat and the trophic and regenerative effects of clinical-grade ADSVF in a 1-day procedure. Our results demonstrate this procedure’s feasibility, its good short-term tolerance profile, and its potential efficacy. In fact, we obtained promising effects with complete clinical and radiological healing of the fistula without any secondary effects. Although the mechanism of action of ADSVF remains poorly understood, findings from prior preclinical and clinical studies in various fields suggest that the combination of angiogenic and anti-inflammatory effects of ADSVF [13, 14] is primarily attributable to the high proportion of MSCs and endothelial progenitor cells; in the reported case, these two types of cells accounted for 38.6% and 4.3%, respectively, of the 22.8 million injected cells. The synergistic effects of these two cell subsets in tissue regeneration and neovascularization have previously been described [15]. As has been consistently reported in the literature, the immunomodulatory properties of ADSVF might be attributable to MSCs, which can reduce the production of pro-inflammatory cytokines and induce the regulatory T-cell phenotype [16–18]. However, these properties have primarily been reported for allogeneic MSCs from healthy donors and should be discussed in the context of an autologous therapy for Crohn’s patients.

## Conclusion

This report highlights the therapeutic potential of a new cellular treatment for Crohn’s patients with refractory perianal fistulas based on the innovative hypothesis that the combined action of ADSVF in association with the trophic characteristics of a microfat graft could be beneficial for this condition. It encourages more extensive follow-up assessments of a greater number of patients to further document the safety and efficacy of the tested procedure. Subsequently, efficacy trials should be considered to assess whether this procedure could significantly improve outcomes for patients with refractory fistulas associated with Crohn’s disease.

## Abbreviations

ADSVF: Adipose-derived stromal vascular fraction
ATMP: Advanced therapy medicinal product
CDAI: Crohn’s Disease Activity Index
GMP: Good manufacturing practice
MRI: Magnetic resonance imaging
MSC: Mesenchymal stem cell
PDAI: Perianal Disease Activity Index
SIBDQ: Short Inflammatory Bowel Disease Questionnaire

## References

[1] Ardizzone S, Porro GB (2007). Perianal Crohn’s disease: overview. Dig Liver Dis.

[2] Marzo M, Felice C, Pugliese D, Andrisani G, Mocci G, Armuzzi A (2015). Management of perianal fistulas in Crohn’s disease: an up-to-date review. World J Gastroenterol.

[3] Dykstra JA, Facile T, Patrick RJ (2017). Concise review: fat and furious: harnessing the full potential of adipose-derived stromal vascular fraction. Stem Cells Transl Med.

[4] Nguyen PS, Desouches C, Gay AM, Hautier A, Magalon G (2012). Development of micro-injection as an innovative autologous fat graft technique: the use of adipose tissue as dermal filler. J Plast Reconstr Aesthet Surg.

[5] Alharbi Z, Opländer C, Almakadi S, Fritz A, Vogt M, Pallua N (2013). Conventional vs. micro-fat harvesting: how fat harvesting technique affects tissue-engineering approaches using adipose tissue-derived stem/stromal cells. J Plast Reconstr Aesthet Surg.

[6] Schwartz DA, Lotus EV, Tremaine WJ (2002). The natural history of fistulizing Crohn’s disease in Olmsted County. Minnesota Gastroenterology.

[7] Eglington TW, Barcaly ML, Gearry RB, Frizelle FA (2012). The spectrum of perianal Crohn’s disease in population based cohort. Dis Colon Rectum.

[8] Scharl M, Rogler G (2014). Pathophysiology of fistula formation in Crohn’s disease. World J Gastrointest Pathophysiol.

[9] Cao Y, Ding Z, Han C, Shi H, Cui L, Lin R (2017). Efficacy of mesenchymal stromal cells for fistula treatment of Crohn’s disease: a systematic review and meta-analysis. Dig Dis Sci.

[10] Panés J, García-Olmo D, Van Assche G, Colombel JF, Reinisch W, Baumgart DC, Dignass A, Nachury M, Ferrante M, Kazemi-Shirazi L, Grimaud JC, de la Portilla F, Goldin E, Richard MP, Leselbaum A, Danese S, ADMIRE CD, Study Group Collaborators (2016). Expanded allogeneic adipose-derived mesenchymal stem cells (Cx601) for complex perianal fistulas in Crohn’s disease: a phase 3 randomised, double-blind controlled trial. Lancet.

[11] Molendijk I, Bonsing BA, Roelofs H (2015). Allogeneic bone marrow derived mesenchymal stromal cells promote healing of refractory perianal fistulas in patients with Crohn’s disease. Gastroenterology.

[12] Garcia-Olmo D, Herreros D, Pascual M (2009). Treatment of enterocutaneous fistula in Crohn’s Disease with adipose-derived stem cells: a comparison of protocols with and without cell expansion. Int J Colorectal Dis.

[13] Serratrice N, Bruzzese L, Magalon J (2014). New fat-derived products for treating skin-induced lesions of scleroderma in nude mice. Stem Cell Res Ther.

[14] Granel B, Daumas A, Jouve E (2015). Safety, tolerability and potential efficacy of injection of autologous adipose-derived stromal vascular fraction in the fingers of patients with systemic sclerosis: an open-label phase I trial. Ann Rheum Dis.

[15] Poitevin S, Cussac D, Leroyer AS (2014). Sphingosine kinase 1 expressed by endothelial colony-forming cells has a critical role in their revascularization activity. Cardiovasc Res.

[16] DelaRosa O, Dalemans W, Lombardo E (2012). Mesenchymal stem cells as therapeutic agents of inflammatory and autoimmune diseases. Curr Opin Biotechnol.

[17] Ghannam S, Pène J, Moquet-Torcy G (2010). Mesenchymal stem cells inhibit human Th17 cell differentiation and function and induce a T regulatory cell phenotype. J Immunol.

[18] Leto Barone AA, Khalifian S, Lee WP, Brandacher G (2013). Immunomodulatory effects of adipose-derived stem cells: fact or fiction?. Biomed Res Int.

